# Unraveling the interplay of neuroinflammatory signaling between parenchymal and meningeal cells in migraine headache

**DOI:** 10.1186/s10194-024-01827-x

**Published:** 2024-07-31

**Authors:** Turgay Dalkara, Zeynep Kaya, Şefik Evren Erdener

**Affiliations:** 1 Departments of Neuroscience and, Molecular Biology and Genetics, Faculty of Science, Bilkent University, Ankara, Turkey; 2 Department of Neurology, Başkent University Faculty of Medicine, Ankara, Turkey; 3 Institute of Neurological Sciences and Psychiatry, Hacettepe University, Ankara, Turkey

**Keywords:** Migraine, Spreading depolarization, Neuroinflammation, Inflammasome, Dural neurogenic inflammation, Pannexin

## Abstract

**Background:**

The initiation of migraine headaches and the involvement of neuroinflammatory signaling between parenchymal and meningeal cells remain unclear. Experimental evidence suggests that a cascade of inflammatory signaling originating from neurons may extend to the meninges, thereby inducing neurogenic inflammation and headache. This review explores the role of parenchymal inflammatory signaling in migraine headaches, drawing upon recent advancements.

**Body:**

Studies in rodents have demonstrated that sterile meningeal inflammation can stimulate and sensitize meningeal nociceptors, culminating in headaches. The efficacy of relatively blood-brain barrier-impermeable anti-calcitonin gene-related peptide antibodies and triptans in treating migraine attacks, both with and without aura, supports the concept of migraine pain originating in meninges. Additionally, PET studies utilizing inflammation markers have revealed meningeal inflammatory activity in patients experiencing migraine with aura, particularly over the occipital cortex generating visual auras. The parenchymal neuroinflammatory signaling involving neurons, astrocytes, and microglia, which eventually extends to the meninges, can link non-homeostatic perturbations in the insensate brain to pain-sensitive meninges. Recent experimental research has brought deeper insight into parenchymal signaling mechanisms: Neuronal pannexin-1 channels act as stress sensors, initiating the inflammatory signaling by inflammasome formation and high-mobility group box-1 release in response to transient perturbations such as cortical spreading depolarization (CSD) or synaptic metabolic insufficiency caused by transcriptional changes induced by migraine triggers like sleep deprivation and stress. After a single CSD, astrocytes respond by upregulating the transcription of proinflammatory enzymes and mediators, while microglia are involved in restoring neuronal structural integrity; however, repeated CSDs may prompt microglia to adopt a pro-inflammatory state. Transcriptional changes from pro- to anti-inflammatory within 24 h may serve to dampen the inflammatory signaling. The extensive coverage of brain surface and perivascular areas by astrocyte endfeet suggests their role as an interface for transporting inflammatory mediators to the cerebrospinal fluid to contribute to meningeal nociception.

**Conclusion:**

We propose that neuronal stress induced by CSD or synaptic activity-energy mismatch may initiate a parenchymal inflammatory signaling cascade, transmitted to the meninges, thereby triggering lasting headaches characteristic of migraine, with or without aura. This neuroinflammatory interplay between parenchymal and meningeal cells points to the potential for novel targets for migraine treatment and prophylaxis.

## Background

Pain typically accompanies an inflammatory response of varying intensity at the site where the nociceptive fibers are activated. Consistent with this observation, migraine headache is proposed to arise from the activation of nociceptive trigeminocervical afferents due to a sterile meningeal inflammatory process [1]. Meningeal headaches triggered by meningeal infections share similarities with migraine headaches, such as the throbbing nature of the headache, photophobia, and phonophobia. However, they are significantly more intense and are associated with evident inflammatory cell reactions in the cerebrospinal fluid (CSF) and gadolinium contrast enhancement in MRI scans [2]. The confined and mild sterile inflammatory process thought to cause migraine headaches either directly originates within the meninges such as in the case of volatile irritants like umbellulone of the headache tree (*Umbellularia californica*) or be triggered by stressful brain perturbations such as aura [3, 4]. However, the mechanism by which benign but stressful brain events underlying migraine prodrome or aura can activate meningeal nociceptors remains unclear. This has led to the hypothesis of episodic central dysregulation in pain pathways as the driving force behind migraine headaches, despite limited evidence [5]. The absence of aura or prodrome in a considerable proportion of migraine attacks has been proposed to support this perspective. Conversely, the effectiveness of anti-calcitonin gene-related peptide (CGRP) antibodies and sumatriptan, which primarily target meningeal nociceptors and trigeminal ganglia located outside the blood-brain barrier (BBB), in treating migraine attacks with and without aura has bolstered the idea that migraine pain originates in the meninges [1, 6]. This is because these agents have limited access to central pathways but effectively target the meningeal nociceptors, despite ongoing controversy [1, 6–8]. Central pathways, however, do play a crucial role in modulating peripheral nociceptive input, as evidenced by significant variability in pain threshold and perception depending on individual’s mental and mood status, notable placebo effect, pain suppression by stimulating the periaqueductal gray, and non-painful auras [5, 6, 9]. Evidence from animal studies indicates that parenchymal neuroinflammatory signaling involving neurons, astrocytes, and microglia, which eventually extends to the meninges [10–18], could potentially link non-homeostatic perturbations in the insensate brain to pain-sensitive meninges. Recent clinical imaging studies have provided further supporting evidence for the presence of parenchymal as well as meningeal inflammation in migraine patients [19, 20].

Cortical spreading depolarization (CSD), the neurophysiological event underlying migraine aura [21], is suggested to potentially trigger headaches, although this remains a topic of debate, particularly due to the challenges associated with observing and directly linking it with headache occurrences in humans [1, 10, 22–24]. Notably, aura manifests contralaterally to the side of the headache, supporting the notion that CSD-induced parenchymal algesic signals may propagate to the overlying meninges, initiating the headache. In other words, disturbances caused by CSD in the occipital cortex result in visual aura primarily on the lateral aspect of the contralateral visual field. Meanwhile, trigeminal nociceptive signals originating from the overlying dura mater enter the brainstem, cross over, and ascend on the contralateral side of the brain, leading to the perception of pain on the contralateral side of the head where the CSD occurred. Thus, a direct spread of CSD-evoked electrophysiological changes (a brief excitatory phase succeeded by several minutes of inhibition) from the cortex to ipsilateral thalamus is challenging to reconcile with the contralateral headache and the temporal gap of 10–60 min between aura and headache onset, as well as the transient nature of these electrophysiological changes. CSD occurring in the visual (V1) or insular cortex has been shown to elicit an early inhibition followed by a delayed facilitation of dura-evoked responses of Sp5C (nucleus caudalis) 2nd order neurons in the rat [25]. This indicates that corticotrigeminal projections have the capacity to modulate dural nociception. However, the clinical results with relatively BBB-impermeable anti-CGRP antibodies and triptans reinforce the idea that the sustained nociceptive activity in migraine is primarily driven by dural neurogenic inflammation [1, 6, 8], which can be modulated by various central mechanisms. These clinical findings also contradict extrapolations suggesting that central facilitatory mechanisms can convert spontaneous non-noxious activity in these areas to the headache of migraine without aura. Conversely, the proposition that “CSD can induce migraine headaches via parenchymal inflammatory signaling, subsequently culminating in sterile meningeal inflammation” is garnering increasing experimental and clinical support [12–20, 26–32] (Table 1). CSD appears to hold promise not only in unraveling the mechanisms behind aura and headache but also in studying the parenchymal neuroinflammatory response to transient brain perturbations that do not result in overt pathology. This form of “neuroinflammation” presents a challenge because much of the existing literature on brain inflammation is centered around disorders characterized by obvious inflammatory reactions, such as those seen in multiple sclerosis (MS) or around amyloid plaques and tumors. In this review, our emphasis will be on delineating the unique characteristics of parenchymal neuroinflammatory signaling induced by CSD, which serves as a model for benign yet impactful brain perturbation capable of precipitating headaches. This focus is a relative departure from our previous review 3 years ago [24], which highlighted meningeal neurogenic inflammation to a comparable extent. Additionally, we aim to present recent updates and underscore advancements in the signaling cascade since its discovery 11 years ago. Critically, we will discuss its potential relevance in understanding migraine headaches.

**Table 1:** CSD induces a parenchymal inflammatory signaling cascade and trigeminal nociception in vivo

Mechanism	Reference*
Trigeminal ganglion and nucleus caudalis activation	*Bolay et al. 2002* [33], *Zhang et al. 2010* [34], *Zhang et al. 2011* [35], *Zhao et al. 2015* [36], *Zhao et al. 2016* [37], *Zhao et al. 2018* [38], *Schain et al. 2018* [39], *Schain et al. 2020* [31], Chen et al. 2023 [30]
Increased middle meningeal artery blood flow (trigeminovascular reflex)	*Bolay et al. 2002* [33], *Karatas et al. 2013* [10], *Schain et al. 2019* [40], *Schain et al. 2020* [31], *Chen et al. 2023* [30]
Pannexin1 activation in neurons	*Karatas et al. 2013* [10], Chen et al. 2017 [16], *Bu et al. 2020* [18], Chen et al. 2023 [30], Dehghani et al. 2023 [27]
Inflammasome formation and caspase-1 activation in neurons	*Karatas et al. 2013* [10], *Chen et al. 2023* [30], *Kaya et al. 2023* [41]
HMGB1 release from neurons	*Karatas et al. 2013* [10], **Takizawa et al. 2016** [12], *Dehghani et al. 2021* [26], Dehghani et al. 2023 [27], *Kaya et al. 2023* [41]
NF-ĸB activation in astrocytes	*Karatas et al. 2013* [10], *Dehghani et al. 2021* [26], *Kaya et al. 2023* [41]
Astrocytosis after repeated CSDs for 4 weeks	Ghaemi et al. 2018 [14]
Induction of proinflammatory enzymes and mediators in cortex/brain in vivo	Caggiano et al. 1996 [42], Miettinen et al. 1997 [43], Yrjänheikki et al. 2000 [44], Jander et al. 2001 [45], Yokota et al. 2003 [46], Thompson et al. 2005 [47], Viggiano et al. 2008 [48], *Karatas et al. 2013* [10], Ghaemi et al. 2018 [14], Chen et al. 2017 [16], Eising et al. 2017 [17], **Takizawa et al. 2020** [13], *Zhao et al. 2021* [29], Volobueva et al. 2022 [32], Chen et al. 2023 [30]
Inactivation of astrocytes prevents CSD-induced nociceptive sensitization	*Zhao et al. 2021* [49]
Activation of pial and dural macrophages, dural dendritic cells	*Schain et al. 2018* [39], *Schain et al. 2020* [31]
Pro-inflammatory microglia activation after multiple (but not single) CSDs	Grinberg et al. 2011 [50], Shibata et al. 2017 [51], Takizawa et al. 2017 [52], Chen et al. 2023 [30]
CSD-induced headache-related behavior	Karatas et al. 2013 [10], **Harriott et al. 2021** [53], Dehghani et al. 2023 [27]
Increased [^11^C]PBR28 uptake in the ipsilateral hemisphere of rats 3 days after multiple CSDs	Cui et al. 2009 [54]
Increased [^11^C]PBR28 uptake in both parenchymal and meningeal regions, and bone marrow in patients having MA attacks in the past 2 weeks	Albrecht 2019 [19], Hadjikhani 2020 [20], Christensen 2022 (review) [55]

* Studies that exclusively use a single CSD are italicized. Studies employing both single and multiple CSDs are marked in bold. Studies utilizing only multiple CSDs and human studies are unmarked

## CSD and headache

CSD is accompanied by the spread of algesic mediators like H^+^, K^+^, ATP, and nitric oxide (NO) from the interstitium into the perivascular and subarachnoid spaces [4, 6, 33]. Although precise molecular mechanisms remain incompletely understood, CSD has been shown to activate perivascular pial nociceptors, as evidenced by the firing of a group of neurons in the trigeminal ganglion and nucleus caudalis concurrently with CSD in the rat, potentially explaining auras coinciding with headaches [34–39, 56]. However, headaches typically start 15–20 min after most migraine auras and, consistent with this clinical observation, the majority of nociceptive units begin firing 15 min after a CSD wave in the rat. The delayed firing of dural nociceptors corresponds with a gradual increase in meningeal artery blood flow, driven by a trigeminoparasympathetic reflex that can be non-invasively recorded through the intact skull, following CSD in rats and mice [10, 33, 40]. Since tissue homeostasis is quickly restored after CSD, this delay has been attributed to the time required for sensitization of trigeminocervical nociceptors [37, 57, 58] and the induction and synthesis of pro-inflammatory enzymes such as cyclooxygenase (COX) 2 and inducible nitric oxide synthase (iNOS) [10] as well as a delayed activation of dural macrophages and dendritic cells, occurring subsequent to the early activation of pial macrophages [39]. Supporting a role for astrocyte endfeet (e.g., for synthesis and release of pro-inflammatory mediators), inactivation of astrocytes abutting pia by fluoroacetate or L-a-aminoadipate has been shown to prevent CSD-induced nociceptive sensitization in the rat [49]. After the initial brief activity of constitutively expressed neuronal nitric oxide synthase (nNOS) and COX1 in cortical interneurons and astrocyte endfeet, the inducible isoforms, iNOS and COX2, can provide high throughput and longer-lasting NO and prostaglandin output [4].

Behavioral tests and electrophysiological recordings from dural afferents have unequivocally demonstrated that a single CSD is sufficient to activate trigeminocervical system and trigger headache-like symptoms in rodents [34–36, 38, 39, 53, 56, 59] (Table 1). Notably, the inflammatory response is intensified following multiple CSDs, leading to the emergence of M1-type inflammatory phenotype in microglia after 24 h [50, 51] (unlike a single CSD exhibiting no M1 phenotype [41, 52]) and perhaps facilitating the detection of headache-related behavior in rodents [10, 53, 60]. While multiple CSDs may serve as an experimental tool to reveal subtle CSD-induced changes, it is essential to recognize that typically, a single CSD precipitates most auras in humans, and multiple CSDs exhibiting a more complex expression profile could be a more suitable model of the inflammatory reaction observed in patients experiencing frequent migraine with aura attacks [13, 14, 50, 51]. Therefore, caution is advised when comparing expression results because not only transcripts but also the cell type that the transcription is altered can vary with the number of CSDs elicited. Accordingly, we will prioritize describing and discussing the neuroinflammatory signaling and transcriptional changes after a single CSD in this review.

Studies have shown that a single CSD induces the opening of neuronal pannexin-1 (Panx1) channels, formation of the inflammasome complex, activation of caspase-1, and subsequent release of interleukin-1 beta (IL-1β) and high mobility group box 1 (HMGB1), which initiate pro-inflammatory NF-ĸB activation in astrocytes [10, 26, 41]. The pro-inflammatory transcription (possibly not limited to the NF-ĸB pathway though not thoroughly explored), leads to the induction of enzymes such as COX2 and iNOS or cytokines such as CCL2, which are normally not appreciably expressed by astrocytes [10, 13, 16, 30, 43, 44, 46, 48, 61–63]. The subsequent release of prostaglandins, NO, and cytokines from the astrocyte endfeet along the glia limitans can participate in activation/sensitization of the pial nociceptors (directly and/or via resident inflammatory cells), thereby contributing to headache generation although precise mechanisms yet to be determined [4, 6, 49]. Supporting these hypotheses with experimental data from rodents, recent positron emission tomography (PET) studies conducted on patients with migraine aura, following the injection of [^11^C]PBR28 (a molecule taken up by glial cells during inflammation), revealed tracer uptake in both parenchymal and meningeal regions [19, 20]. Intriguingly, tracer uptake was simultaneously registered in the affected occipital (aura) cortex and the overlying dura in some patients. This finding supports the concept that CSD-induced parenchymal inflammatory signaling can propagate to the meninges, inducing meningeal inflammation and consequently, headache in patients as suggested by experimental studies [20]. The enhanced tracer uptake in the visual cortex overlying meninges was also found to extend to the adjacent bone marrow [20, 55]. As elucidated recently, skull channels provide direct communication between the meninges and the skull bone marrow [64]. In case of overt inflammation such as bacterial meningitis, bone marrow presents myeloid cells that migrate through these channels and initiate local inflammatory response [65]. The involvement of bone marrow in various neurological disorders such as MS or Alzheimer’s disease is increasingly being recognized in both experimental models and patients [66, 67]. The surprising finding of tracer uptake extending to the bone marrow in migraine with aura patients suggests that myeloid cells may contribute to inflammation and reinforces the significance of sustaining dural inflammation for headache generation in migraine.

## Neuronal stress sensors – Pannexin1 channels

**Fig. 1:**
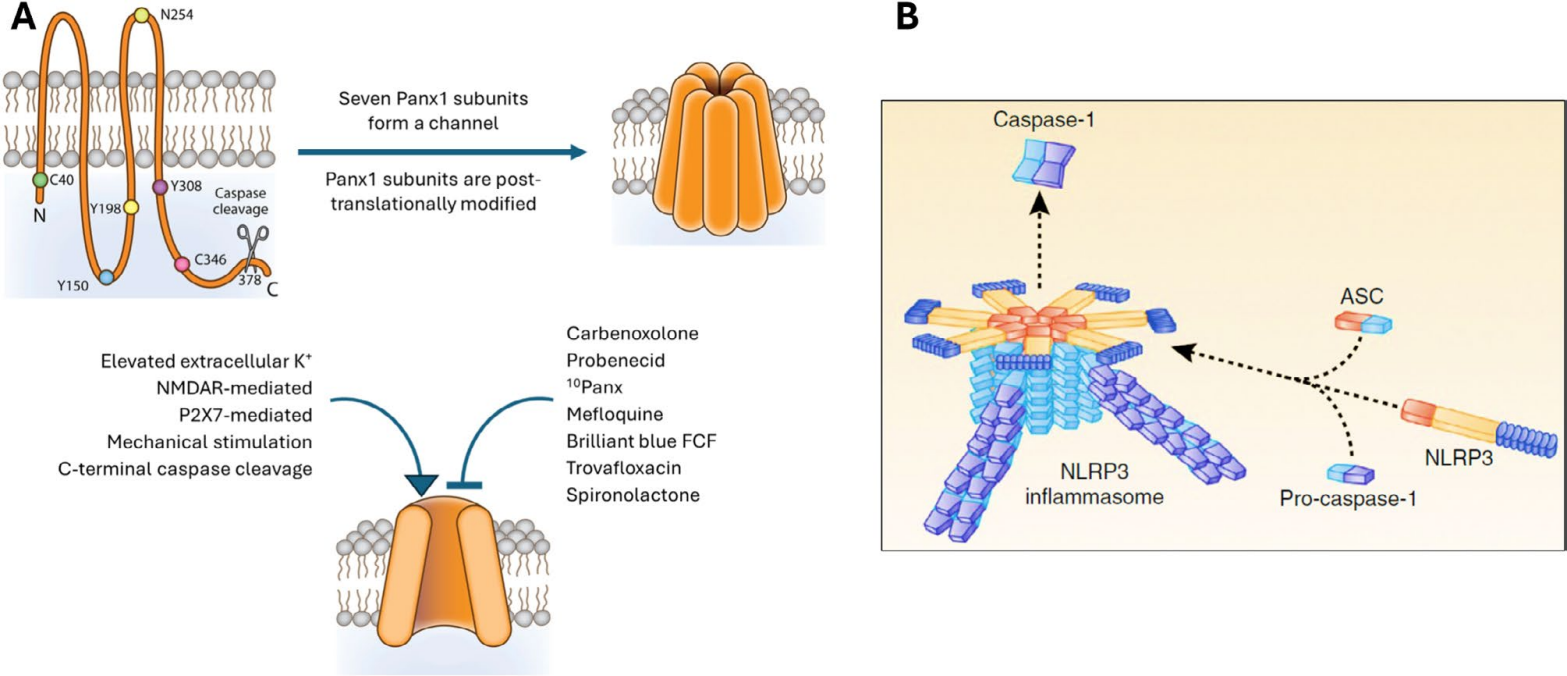
**(A)** Pannexins are heptameric transmembrane proteins that form large-pore ion channels. Subunits undergo post-translational modifications; for instance, Src-family kinases phosphorylate Y308, promoting pore opening, while caspase cleavage at the 378th amino acid leads to permanent channel opening and cell death under pathological conditions. During CSD, Panx1 channels in neurons can be activated by high extracellular K^+^, glutamate, and intracellular Ca^2+^ concentration, depolarization and NMDA receptor stimulation as well as by Src-family kinases. **(B)** CSD-induced NLRP3 inflammasome complex formation is a downstream event triggered by Panx1 channel activation. Inflammasome assembly serves as an initial step in inflammatory conditions, facilitating the processing of pro-inflammatory mediators into their active forms. This assembly involves the clustering of node-like receptors around a central hub which is facilitated by the recruitment of an adapter molecule containing a caspase recruitment domain (ASC). Pro-caspase-1 binding to this complex dimerizes and undergoes self-cleavage, releasing active caspase-1. Reproduced from [82] and [83] with permission.

Pannexins are heptameric transmembrane proteins that host a large-pore ion channel [68] (Fig. 1). Within the nervous system, both Panx1 and Panx2 are identified. Panx1 exhibits broad expression across excitatory and inhibitory neurons, as well as oligodendrocytes, astrocytes, and microglia [69]. In neurons, its primary localization is at the postsynaptic membrane [70]. Panx1 serves as a modulator of glutamatergic transmission and acts as a sensor for stressful pro-inflammatory conditions in the brain by triggering inflammasome formation and downstream inflammatory signaling [71–73].

Panx1 channels can be activated by various signals present during CSD such as high extracellular K^+^, glutamate, and intracellular Ca^2+^ concentration [74], depolarization and N-methyl-D-aspartate (NMDA) receptor stimulation [75], and plasma membrane stretch (e.g. spine swelling). They may also be permanently opened by cleavage of the C-terminal region during apoptosis, contributing to cell death under pathological conditions [76]. When Panx1 opens in a large-conductance state, its nonselective ion channel becomes permeable to molecules up to 900 Da, allowing considerable K^+^ and ATP efflux [73, 77]. This unique property enables the detection of Panx1 opening using membrane-impermeant fluorescent dyes smaller than 900 Da, like propidium iodide or YoPro-1. Thus, membrane-impermeable dyes can enter a cell through large channel openings [78, 79]. This feature has been crucial in revealing CSD-induced Panx1 activity in the mouse and rat brain [10, 18]. Because CSD-induced perturbations last approximately 2 min, Panx1 opening is transient as shown by propidium iodide influx to neurons [10]. Nevertheless, this timeframe proves adequate for promptly initiating inflammasome formation (Fig. 1B) and activating caspase-1 in neurons, as detected 5 [10] and 15 [30] minutes after a single CSD induced by pinprick or optogenetically by two independent laboratories. This observation holds true for both male and female mice, which is crucial to emphasize because migraine prevalence is significantly higher in females, and females experience autoimmune and autoinflammatory diseases more frequently [80, 81]. However, most experimental research on migraine has historically focused on male animals, though this trend is changing. Notably, NLRP3 has recently been identified as the NLRP subtype responsible for forming the inflammasome in neurons. Its inhibitor MCC950 effectively suppressed caspase-1 cleavage induced by CSD [30].

Membrane-impermeant fluorescent dyes can also enter through P2x7 receptor (P2rx7) channel pore, which can open in a large conductance state, possibly induced by extracellular ATP reaching high levels during CSD [84]. Notably, the P2x7/Panx1 pore inhibitor A438079 [16] as well as disruption of the interaction of P2x7 receptor with Src family kinases by TAT-P2x7 [85] have been shown to reduce the increase in IL-1β expression after CSD. However, the location of P2rx7 channels initiating the inflammatory transcription, whether on neurons or glial cells, remains currently unknown as astrocytes and microglia also harbor Panx1 and P2rx7 as well as Src-family kinases interacting with them [86]. Although increased IL-1β expression after CSD (correlated with the number of CSDs [16]) has long been recognized [45, 47], this represents a distinct process (transcriptional expression) compared to the cleavage and activation of the constitutively present pro-IL-1β by caspase-1 in neurons following the activation of Panx1 channels and inflammasome formation, as discussed earlier.

Strongly supporting the notion that CSD-induced propidium iodide influx to neurons occurs through large channel opening of the neuronal Panx1, this was prevented not only by the non-selective Panx1 blockers carbenoxolone and probenecid but also the selective inhibitor ^10^Panx peptide [10]. Additionally, RNAi-mediated suppression of Panx1 expression proved to be a successful strategy in inhibiting this process [10]. In line with the involvement of Panx1 channels, Panx1 mRNA in the cortex was reportedly upregulated following a single CSD [32] as well as after synaptic metabolic stress causing Panx1 activation [87]. Of note, P2x7/Panx1 channels present in glial cells may also facilitate CSD generation and propagation, for instance by releasing K^+^, as suggested by studies using P2x7/Panx1 channel inhibitors in addition to their role in inflammatory signaling [16].

The exact mechanism of how neuronal Panx1 channels open in a large-conductance state after CSD has not been thoroughly investigated. In addition to factors such as high extracellular K^+^ and neuronal swelling [10, 87], it has been proposed that intense stimulation of NR2A type NMDA receptor subunits by high extracellular glutamate and strong depolarization during CSD activates Src-family kinases [18]. These kinases, in turn, phosphorylate Y308 near the intracellular C-terminal, thereby promoting the opening of Panx1 channels [18]. Indeed, the TAT-Panx308 peptide, which inhibits Y308 phosphorylation by Src-family kinases, has been shown to prevent CSD-induced HMGB1 release [27]. Similarly, the Src-family kinase inhibitor, PP2, or the NR2A–receptor antagonist, NVP–AAM077, when perfused into cerebral ventricles of rats prior to CSD induction, attenuated CSD-induced Panx1 activation in cortices [18].

**Fig. 2:**
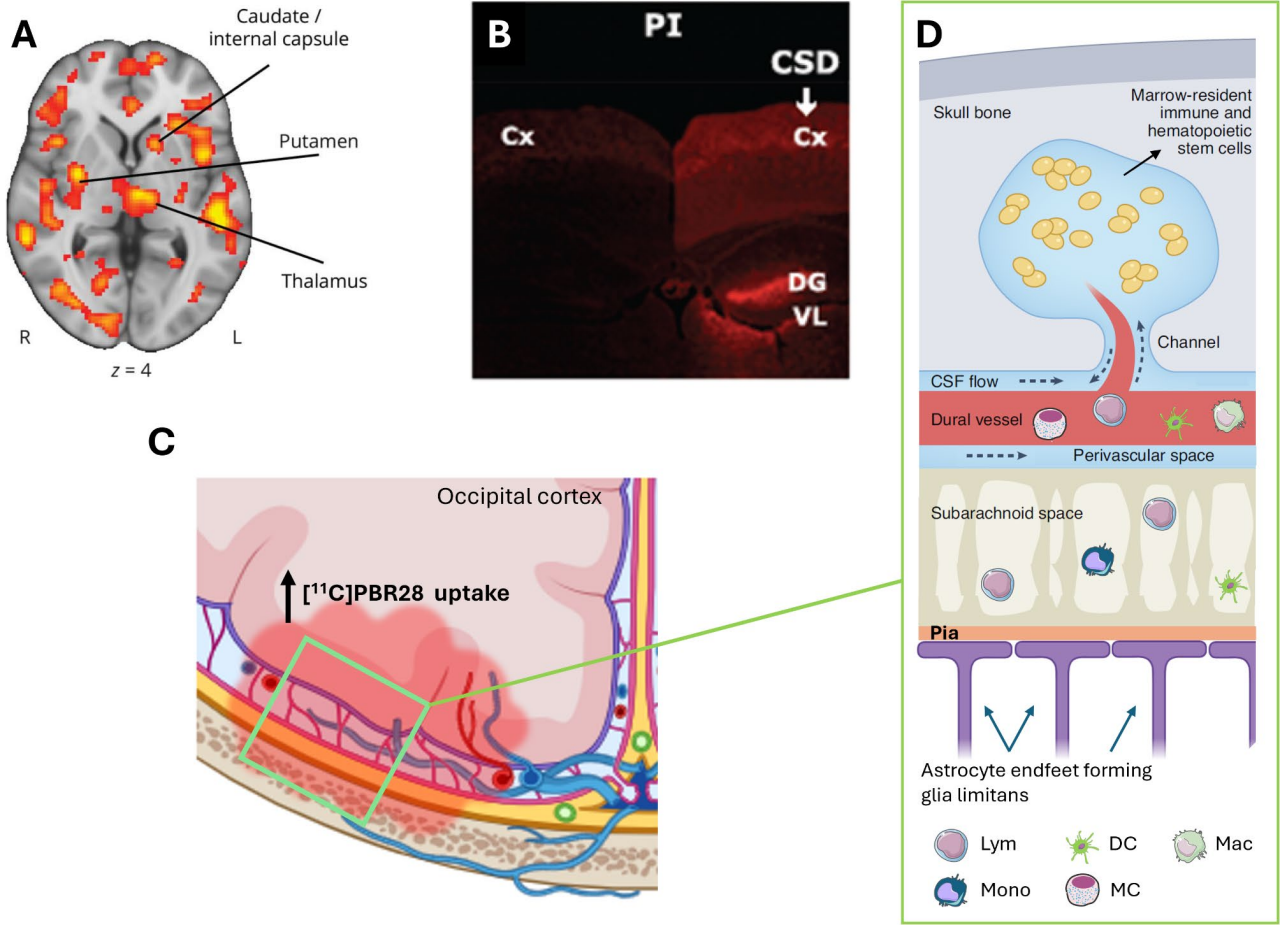
CSD-induced inflammatory activity propagates through the brain, meninges, and skull. **A**. PET studies utilizing inflammation markers revealed bi-hemispheric cortical as well as subcortical inflammatory tracer uptake in patients suffering from frequent migraine with aura attacks. **B**. Consistent with clinical observations, Panx1 activity, monitored by propidium iodide (PI) influx to neurons (red fluorescence), was not confined to the cortex (Cx) ipsilateral to CSD but was also evident in the contralateral cortex and subcortical structures such as the dentate gyrus (DG) in the mouse brain. **C**. Simultaneous tracer uptake ([^11^C]PBR28) was observed in the affected occipital cortex responsible for generating the aura and the overlying dura, extending to the adjacent bone marrow. These findings suggest that myeloid cells may also contribute to inflammation in addition to the inflammatory mediators released from astrocyte endfeet and dural cells **(D)**, thus underscoring the significance of sustained dural inflammation in migraine headache generation. Lym: lymphocyte, DC: dendritic cell, Mac: macrophage, Mono: monocyte, MC: mast cell. Reproduced from [10, 19, 91] with permission. Illustrations were created using BioRender.com and Servier Medical Art (http://www.servier.com).

Interestingly, neuronal Panx1 activation as monitored by propidium influx was not limited to the cortex ipsilateral to CSD. It was also observed in the contralateral cortex and subcortical structures such as the dentate gyrus [10, 88] (Fig. 2B). Subsequent validation of this observation included the demonstration of widespread HMGB1 release from neurons and NF-ĸB activation in astrocytes in cortical and subcortical areas of both hemispheres [26]. These effects were less intense in the contralateral hemisphere. Importantly, these experimental observations conform with PET findings that revealed bi-hemispheric cortical as well as subcortical inflammatory tracer uptake in patients suffering from frequent migraine with aura attacks [19, 20] (Fig. 2A). The mechanisms underlying the spread of this phenomenon and its potential association with bilateral headaches following unilateral aura remain unclear. Notably, the significant propidium iodide uptake in dentate gyrus granular neurons, in contrast to neighboring CA sector pyramidal neurons, suggests a propagation via axonal volleys from the entorhinal cortex rather than gray matter or interstitium continuity. These volleys typically fire at the onset of CSD wave before depression of electrical activity [89]. The heightened excitatory firing, akin to observations during epileptiform discharges, has the potential to activate Panx1 channels due to the overactivation of NMDA receptors and rise in extracellular K^+^ [75, 90]. In support of this notion, when NMDA receptors were inhibited by locally applied MK801 to the cortex contralateral to the site where CSD was generated, HMGB1 release was suppressed in the contralateral (non-CSD) cortex, without any discernible impact on the CSD occurring on the ipsilateral side [26]. Likewise, in familial hemiplegic migraine type 1 mice exhibiting enhanced glutamate release due to a knock-in S218L missense mutation in α1A subunit of presynaptic CaV2 (but not in R192Q knock-in exhibiting less severe phenotype [26]), HMGB1 release in the contralateral cortex was increased [27].

While studies involving CSD have been crucial in uncovering and exploring parenchymal inflammatory signaling initiated by the activation of neuronal Panx1 channels, a lingering question remains about whether the same pathway could be triggered by transient neuronal disturbances other than CSD. This potential mechanism could offer insights into migraine without aura arising from brain perturbations, such as sleep deprivation, distinct from migraine without aura caused by factors directly activating meningeal nociceptors. Indeed, experiments creating synaptic stress by inhibiting glycogen use have shown the opening of neuronal Panx1 channels, caspase-1 activation, and the release of HMGB1 in the absence of CSD in mice [87, 92]. This occurrence is attributed to the essential role of glycosyl units derived from glycogen in astrocyte processes, fueling astrocytic uptake of glutamate and K^+^ during rapidly escalating intense neuronal activity. Migraine triggers, including sleep deprivation or acute psychological stress, induce transcriptional changes in astrocytes [92]. Some of these changes promote glycogen synthesis in astrocyte processes over its utilization, potentially jeopardizing the clearance of glutamate and K^+^ during high-frequency/prolonged neuronal activity [92]. Consequently, based on experimental evidence, we can hypothesize that migraine triggers hold the potential to activate the parenchymal inflammatory signaling, leading to headaches without the necessity of CSD and, consequently, without the occurrence of aura.

Owing to its upstream role in the initiation of inflammation, Panx1 is being considered as a therapeutic target for treating inflammatory diseases such as rheumatoid arthritis. The aforementioned findings also highlight it as a potential target for prophylaxis of migraine with aura and, perhaps, migraine without aura. In experimental settings, it is possible to inhibit Panx1 or purinergic receptor activity with non-selective pharmacological agents like carbenoxolone, probenecid, mefloquine, flufenamate [93], spironolactone, nitric oxide donors (by S-nitrosylation at Panx1 C346) [79], quinolones and brilliant blue FCF or G to name a few among a growing number of agents [94] (Fig. 1A). Additionally, selective peptides such as ^10^Panx and specific conventional or mini antibodies can be employed [93]. While carbenoxolone, probenecid, mefloquine, spironolactone, floxacins are clinically registered drugs and brilliant blue G is a commercially used candy additive [79], there are no published reports on their potential effect on migraine at the clinically used doses. Notably, flufenamate was used in the past as a nonsteroidal anti-inflammatory drug for treating menstruation-related migraine [95]. However, the question of whether these agents can achieve effective concentrations in the cortex to inhibit neuronal Panx1 channels after systemic administration of clinically used doses (e.g. carbenoxolone is poorly BBB permeable [96]) and whether any unwanted side effects could overshadow (e.g. spironolactone is 1000-times more potent in blocking mineralocorticoid receptors [97]) their migraine prophylactic action remains unclear (see [93] for review). Just like Panx1 inhibitors, there is growing consideration for inflammasome and caspase-1 inhibitors as potential therapeutic targets for treating inflammatory diseases (reviewed in [98, 99]). This exploration may pave the way for clinical trials involving promising candidates in the treatment of migraines. Of note, the anti-inflammatory agents developed may not only address parenchymal inflammatory signaling but also potentially suppress dural neurogenic inflammation. As a result, these agents could serve a dual purpose by being utilized not only in migraine prophylaxis but also in the treatment of acute migraine attacks. However, it’s important to note that effective doses for the brain and meninges could vary significantly due to factors such as the BBB permeability and the differing abundance of targets to be inhibited. Higher doses could potentially lead to unwanted effects. Additionally, inhibiting widely expressed upstream targets such as inflammasomes carries the risk of undesired immunomodulation, a common concern in drug development, for instance, for rheumatic diseases.

## Proinflammatory mediators released from neurons

The CSD-induced formation of the NLRP3 inflammasome complex represents a downstream event triggered by the activation of Panx1 channels in neurons (Fig. 1B). Inflammasome formation serves as a common initial step in various inflammatory conditions, establishing molecular machinery for processing of pro-forms of proinflammatory mediators into their active forms. The assembly of an inflammasome complex involves the clustering of node-like receptors (NLRs) around a hub when detecting pathogen- or cellular damage-associated signals in the cytoplasm. This clustering is completed by the joining of an adapter molecule containing a caspase recruitment domain [100, 101]. Pro-caspase-1 binding to this complex dimerizes and undergoes self-cleavage, releasing active caspase-1. Subsequently, this active enzyme mediates the cleavage of pro-IL-1β and pro-IL-18, generating active IL-1β and IL-18. Besides the formation of the NLRP3 inflammasome complex after CSD and the emergence of the cleaved form of caspase-1 mentioned above, the released active IL-1β from neurons has been identified in CSF [10] and brain rinsing solution [30]. It is worth noting that a technical drawback in studying IL-1β lies in the fact that the available antibodies typically recognize both the pro and cleaved forms of IL-1β. Overcoming this limitation, the detection of secreted active form in CSF provides valuable insights, albeit with technical challenges associated with collecting CSF from small rodents. The release of IL-18 along with IL-1β is likely to occur as generally observed in other cells [102, 103], although its role in the context of CSD has not been explored. In addition to cleavage of existing pro-IL-1β in neurons and release of IL-1β, an increase in IL-1β expression has been detected as early as 10 minutes after a single noninvasive (optogenetically triggered) CSD in the mouse [13] and after potassium chloride or microinjury-induced single CSD in the rat [32]. Multiple CSDs cause a more robust increase in IL-1β transcription, accompanied by the expression of several other pro-inflammatory genes [13, 16]. This transcriptional response was reduced in IL-1 receptor-1 knockout mice, suggesting that it was initiated by IL-1β released from neurons [13]. Supporting a neuronal origin for this inflammatory activity, ^10^Panx and NLRP3 inhibitor MCC950 ameliorated SD-induced upregulation of IL-1β transcription [30].

Parenchymal IL-1β production could also play a significant role by triggering meningeal nociceptor activation in migraine without aura (i.e. without CSD) [87, 92]. Indeed, migraine without aura attacks are seen in patients with cryopyrin-associated periodic syndromes (CAPS), where IL-1β is overproduced due to mutations in the NLRP3 inflammasome. Further supporting the involvement of parenchymal inflammatory signaling in migraine without aura, elevated levels of IL-1β, prostaglandin E2, tumor necrosis factor-α (TNF-α), IL-6, and nitrite were detected in the internal jugular vein (which primarily drains the brain parenchyma but not the meninges) within the first hour of a migraine without aura attack [61, 104–106]. Interestingly, migraine attacks in CAPS patients are suppressed with the IL-1 receptor antagonist anakinra [107–109]. Considering the poor BBB penetrance of anakinra, its main site of action could be the dura as IL-1β activates meningeal nociceptors and increases their mechanosensitivity [110, 111]. However, these observations reinforce the idea that agents antagonizing the action of IL-1β could be used in migraine prophylaxis and attack treatment if not limited by potential side effects.

Inflammasome activation is also associated with the translocation of HMGB1 from the nucleus to the cytoplasm [112, 113]. HMGB1, a non-histone protein that binds to DNA, is abundantly expressed in nearly all cells and serves various nuclear functions [114]. However, it transforms into a proinflammatory mediator upon release into the extracellular medium, akin to other alarmin proteins such as IL-33 or S100β [115]. HMGB1 passively leaks from necrotic or damaged cells but it can also be actively transported out of the cell after an inflammatory stimulus such as cell swelling, tissue injury, or infection [116, 117]. In such cases, its three-dimensional structure changes by acetylation, phosphorylation, or methylation of different amino acids [117]. This structural alteration exposes the nuclear export signal necessary for the translocation of HMGB1 from the nucleus to the cytoplasm. HMGB1 can activate various inflammatory pathways including NF-κB in nearby cells expressing receptors for advanced glycation end products (RAGE) and toll-like receptors (TLRs) [117, 118].

**Fig. 3:**
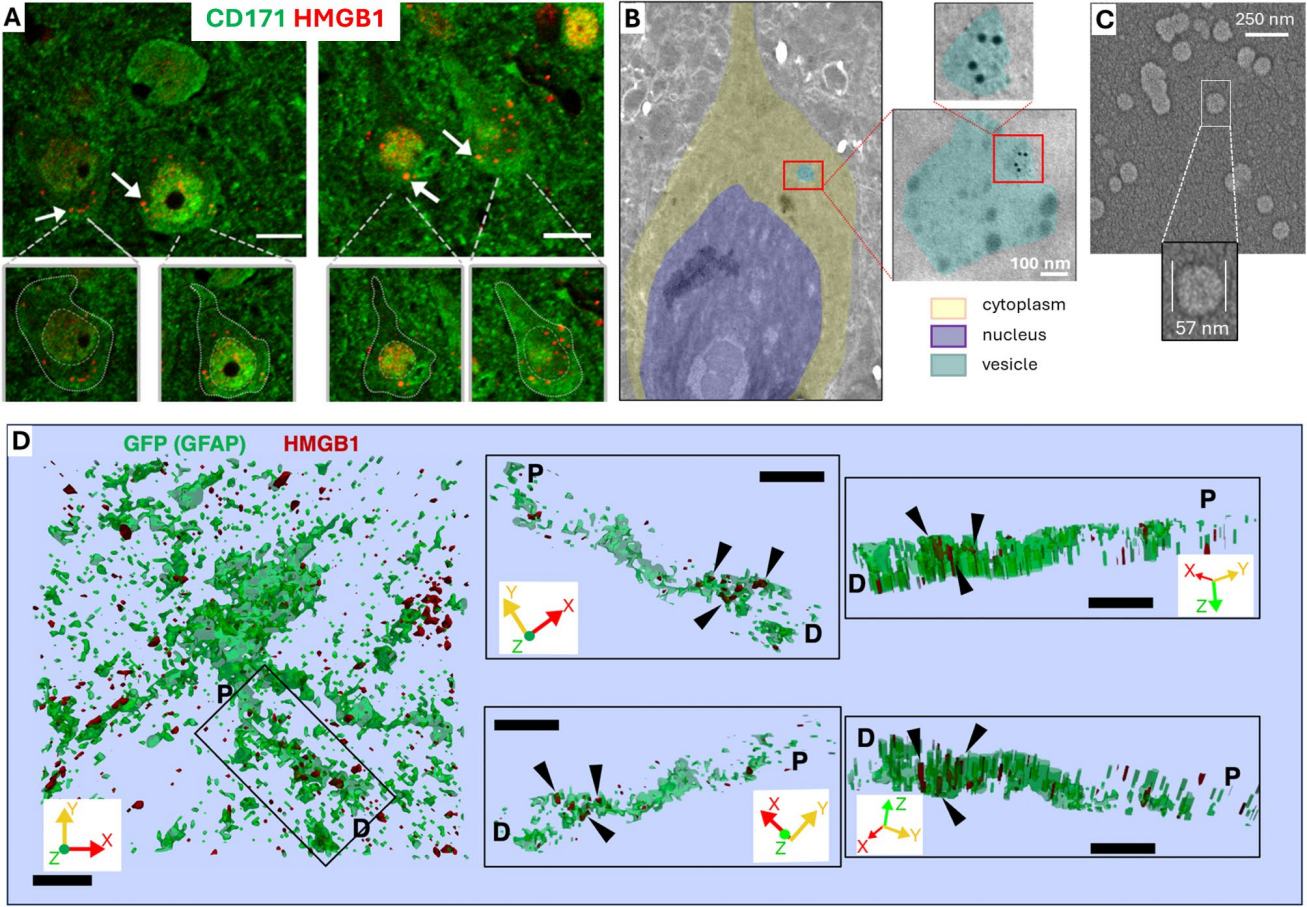
A single CSD triggered by pinprick causes release of HMGB1 from neurons within small EVs, which are subsequently taken up by astrocyte processes. **(A)** Immunolabeling reveals numerous HMGB1-positive puncta (red, marked by white arrows) in the cytoplasm surrounding the nuclei of cortical neurons, identified by CD171 immunolabeling (green). Insets below delineate the boundaries of neuronal cytoplasm and nucleus, emphasizing the distribution of the puncta. Shedding of HMGB1-labeled puncta from cells, with varying degrees of nuclear HMGB1 immunopositivity loss, is observed as early as 15 min post-CSD. Puncta near the nuclei (white arrows) suggest HMGB1 release within vesicles. Images are maximum projections of confocal z-stacks. Scale bars: 10 μm. **(B)** Electron microscopic (EM) images of a neuron depict a multivesicular body (light blue) containing several small EVs, one of which carries gold nanoparticles marking HMGB1 1-hour post-CSD. **(C)** Transmission EM image of EV suspension isolated from mouse brain, predominantly having a size compatible with exosomes. **(D)** 3D surface reconstruction of a GFP‐positive astrocyte and its process shows that HMGB1-immunopositive puncta (black triangles) are located inside the process. The black rectangle on the left panel indicates the HMGB1‐immunopositive process that is visualized on the right panels from different angles in 3D. P and D denote the proximal and distal ends of the process, respectively. Scale bars: 2 μm. X, Y, and Z axes of the volume are shown for orientation. Reproduced from [41] under Creative Commons Attribution 4.0 International License (http://creativecommons.org/licenses/by/4.0/)

Depending on brain region, approximately 40–80% of the neuronal nuclei exhibit loss of HMGB1 immunoreactivity immediately after a single CSD, whereas glial nuclei remain unaffected [10, 26] (Fig. 3A). Optogenetically-induced CSD results in comparable HMGB1 release to pinprick- or Potassium chloride-induced single CSDs, confirming that HMGB1 release is specifically triggered by CSD but not experimental injury [26, 27, 30, 41](Table 1). A recent study demonstrates that, after a single CSD induced optogenetically or by pinprick, HMGB1 is released from neurons within extracellular vesicles (EVs), predominantly having a size compatible with exosomes [41] (Fig. 3B, C). This is in line with the fact that HMGB1 molecule does not have a leader peptide sequence to cross the plasma membrane by conventional protein secretion mechanisms [119, 120]. Interestingly, released exosomes are promptly taken up by astrocyte processes enveloping neuron soma (Fig. 3D), leading to NF-ĸB activation in these cells, which was previously shown to be suppressed by knocking down HMGB1 expression or by inhibiting HMGB1 activity with anti-HMGB1 antibodies or BoxA fragment of HMGB1 applied before CSD [10]. In contrast, microglia do not internalize HMGB1-bearing EVs and exhibit neither NF-ĸB activation nor the conventional inflammatory phenotype even 24 h after CSD [41]. After multiple CSDs, some of the released HMGB1 leaks into CSF, reaching detectable levels with Western blotting [10]. As a result, a slight reduction in HMGB1 levels in cortex extracts can be observed 2–3 h after multiple [12, 30], but not single, CSDs giving the impression that only multiple CSDs could cause HMGB1 release [12]. Consequently, the most reliable parameter to show CSD-induced HMGB1 release appears to be the loss of nuclear HMGB1 immunoreactivity detected by immunohistochemistry [10, 12, 30].

## Astrocyte and microglia activation and NF-κB pathway

**Fig. 4:**
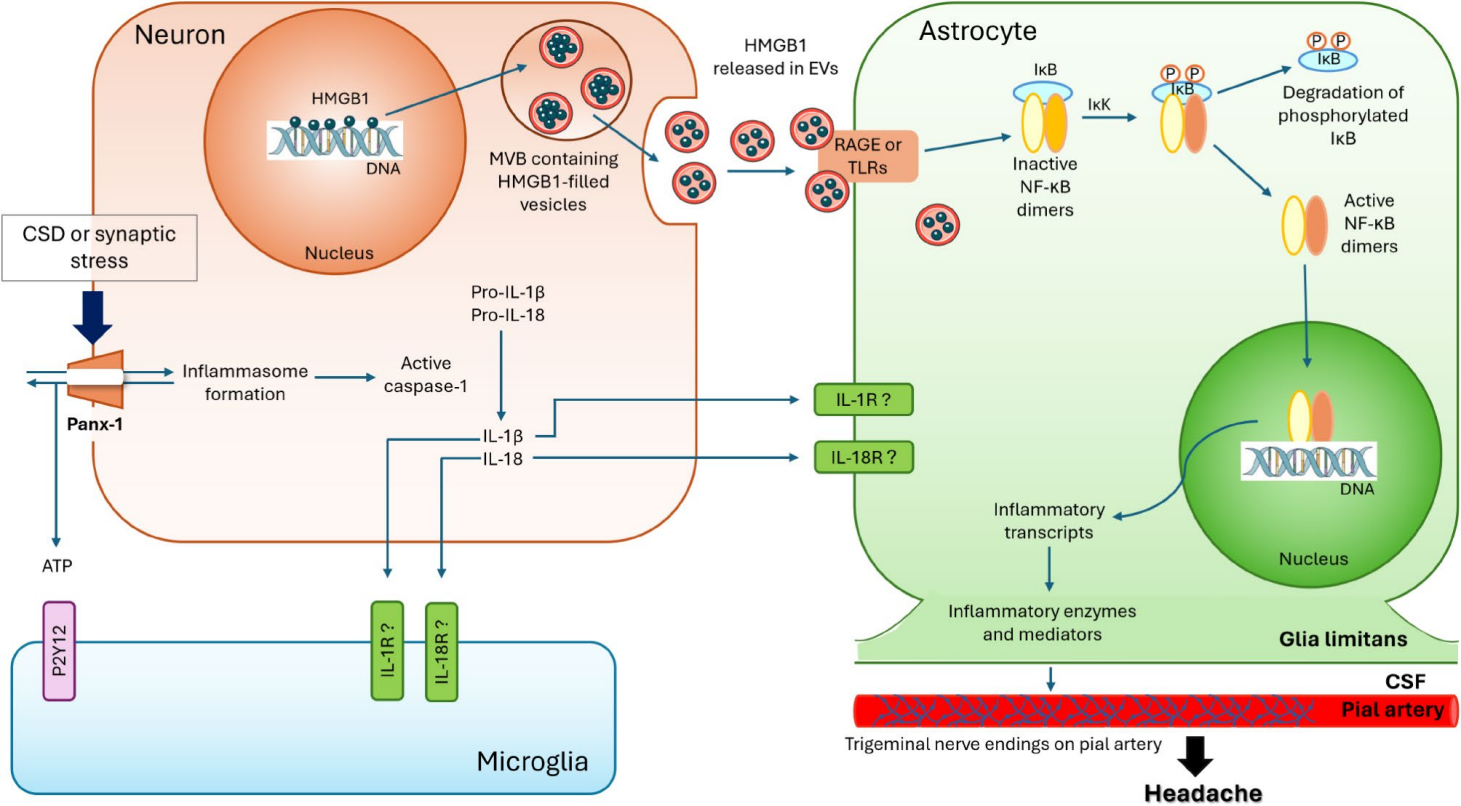
A single CSD or synaptic stress induces the opening of neuronal Panx1 channels, formation of the inflammasome complex, activation of caspase-1, and subsequent release of IL-1β and HMGB1, which induce translocation of NF-ĸB pairs to the nucleus to initiate pro-inflammatory transcription in astrocytes. The pro-inflammatory transcription in astrocytes leads to the induction of enzymes such as COX2 and iNOS or cytokines such as CCL2. The subsequent release of prostaglandins, NO, and cytokines from the astrocyte endfeet along the glia limitans can activate/sensitize the pial nociceptors, thereby contributing to sustaining headache. ATP release from Panx1 channels attracts microglia processes that continuously survey the spines via P2Y12 receptors to repair injured spines. Illustrations were created using Servier Medical Art (http://www.servier.com)

Both astrocytes and microglia express receptors that can respond to HMGB1. However, recent findings reveal that HMGB1 released in EVs selectively initiates an inflammatory signaling in astrocytes without activating the NF-κB system in microglia after a single CSD (Fig. 4). Microglia are known to exhibit an inflammatory phenotype only after multiple CSDs and with a 24-hour delay, dependent on TLR2/4 [52]. The latter may be more relevant to inflammatory activity in patients experiencing frequent migraine with aura attacks, rather than the parenchymal inflammatory signaling mediated by astrocytes after a typical single aura. In addition, the glia limitans formed by astrocyte endfeet, covers the whole cortical surface and perivascular spaces, providing a large surface area for transporting proinflammatory mediators to the pial nociceptors as well as to the CSF. This creates an opportunity for direct access to dural nociceptors in addition to their activation via pial collaterals [4]. Even when microglia are in an active proinflammatory state, their cytokines released into the interstitium seem to reach CSF in perivascular and subarachnoid spaces through a tight extracellular space [121]. Accordingly, astrocytes covering the entire cortical surface and forming an elaborate syncytium among themselves are in a prime position to facilitate communication of inflammatory signaling between the brain parenchyma, pia, and CSF. Conversely, microglia continuously survey the spines and dendrites with their processes, participating in recycling and repairing synapses and, when injured irreparably, in removing them [51, 122–124]. In fact, a recent study has demonstrated that neuronal swelling-induced by opening of Panx1 channels leads to ATP release, which attracts microglia processes via P2Y12 receptors exclusively expressed in microglia [125]. These observations raise the possibility that while astrocytes stimulated by HMGB1 activate an inflammatory signaling cascade to excite pial nociceptors, microglia promote repair programs involving the expression of cytoprotective cytokines.

While the inflammatory response is inherently complex and entails multiple pathways, the translocation of NF-κB subunits to the nucleus suggests that transcriptional NF-κB activity in astrocytes likely plays a role for orchestrating this process, from the secretion of pro-inflammatory algesic signals to the CSF, to the anti-inflammatory resolution phase. The latter activity may contribute to the termination of dural neurogenic inflammation and alleviating headache. The NF-κB transcription factor family operates by combining p65, cRel, RelB, p52, and p50 subunits in pairs. Depending on the specific subunit pairs, NF-κB either promotes the expression of pro-inflammatory molecules or anti-inflammatory ones. For instance, while the p65:p50 pair promotes the expression of inflammatory genes such as iNOS, COX2, and TNF-α, the cRel-containing pairs induce the expression of anti-inflammatory/survival genes such as transforming growth factor beta (TGF-β) and Bcl-x [126–128]. The relative abundance of transcripts for these pairs determines the overall behavior of the nucleus [129, 130]. Furthermore, NF-κB pairs can influence the transcription of various NF-κB subunits and inhibitory-kappa B (IκB), which plays a role in terminating the transcriptional activity of NF-κB pairs. Our recent studies have revealed that pro-inflammatory NF-κB p65:p50 pairs, as well as anti-inflammatory cRel:p65 pairs are both translocated to astrocyte nuclei shortly after CSD [131]. Interestingly, however, 24 h after CSD, the nuclear p65:p50 pairs disappear while cRel:p65 persist, consistent with a shift from pro-inflammatory to anti-inflammatory transcriptional activity in astrocytes. One of the steps that terminates NF-κB activation is the translocation of IκB to the cell nucleus. Consistent with this, we detected IκB in astrocyte nuclei along with p65 and cRel shortly after CSD. Microglia may also contribute to the resolution of the parenchymal inflammatory signaling by switching to an anti-inflammatory phenotype, however, this remains to be investigated [132].

## Clinical outlook and conclusions

Advancements in neuroimaging techniques are promising to be able to directly assess the presence of the mechanisms discussed above in migraine patients. Particularly encouraging is the detection of the meningeal uptake of the inflammatory tracer [^11^C]PBR28 over the occipital cortex, exhibiting parenchymal uptake in patients suffering from migraine with visual aura [20] (Fig. 2). [^11^C]PBR28 PET may also provide insight into the relationship between inflammatory signaling and headache in secondary headache disorders such as post-seizure headache. [^11^C]PBR28 exploits its high affinity against the 18 kDa translocator protein (TSPO) in the outer mitochondrial membrane, an inflammation-specific biomarker in activated glial cells. TSPO-PET imaging is increasingly being utilized for various clinical populations to disclose neuroinflammatory involvement. For example, in patients with chronic neurodegenerative disorders such as amyotrophic lateral sclerosis and Alzheimer’s disease, inflammatory glial activation in the central nervous system (CNS) has been demonstrated starting from the early stages [133, 134]. Despite inconsistent results with [^11^C]PBR28 [135], another TSPO ligand, [^18^F]FEPPA, showed a notable increase in glial uptake in patients with depression in relevant regions like the anterior cingulate cortex and hippocampus [136]. These discrepancies underscore the ongoing need for improved PET ligands, as TSPO signals can be confounded by variable binding affinities depending on TSPO gene polymorphisms, issues with TSPO binding specificity, and their in vivo metabolic profiles. Next-generation tracers with enhanced TSPO binding features are in active development [137]. If successful, these improved tracers can play a pivotal role in resolving some of the controversies surrounding the role of meningeal neurogenic inflammation and parenchymal inflammatory signaling in migraine. Moreover, it is worth noting that, inflammatory glial activation in the brain and the spinal cord has also been shown with [^11^C]PBR28 PET in chronic pain conditions other than migraine, such as chronic low back pain [138, 139] and fibromyalgia [135], suggesting a shared neuroinflammatory element across a heterogeneity of pain-related conditions [140].

In conclusion, neuroinflammatory mechanisms are garnering increasing attention in CNS disorders. The hypothesis of neuroinflammatory signaling following transient perturbations such as CSD or synaptic metabolic stress has received considerable experimental support over the past decade. The demonstration of inflammatory tracer uptake in brain parenchyma as well as the meninges in migraine with aura patients aligns with these experimental findings, reinforcing the notion that inflammatory mechanisms may play a pivotal role in headache generation after brief perturbations and in sustaining the pain. Available evidence suggests that astrocyte endfeet covering the brain surface and perivascular spaces could serve as an extensive interface for transducing parenchymal inflammatory signaling to neurogenic inflammation in the meninges, where pial nociceptors detect the algesic signals and activate the dural nociceptors via collaterals, resulting in release of peptides such as CGRP [6]. These peptides stimulate dural inflammatory cells, inducing secretion of algesic and inflammatory mediators, thereby contributing to sustaining inflammation and nociceptive activity, hence, headache [4, 6, 24]. Supporting the role of dural neurogenic inflammation in headaches, various rodent models have shown that the application of inflammatory substances (e.g., complete Freund’s adjuvant, inflammatory soup) onto the dura causes headache-like behaviors such as peri-orbital allodynia, facial grooming and scratching, along with activation of trigeminal ganglion and nucleus caudalis neurons, as well as trigeminal ganglion satellite cells [34, 35, 141–145]. Additionally, these models exhibit pain-related general behaviors such as freezing and reduced locomotor activity [141]. Of note, the primary factors contributing to female vulnerability for migraine, estrogen and testosterone indeed influence the pain processing networks. Testosterone and estradiol exhibit anti-nociceptive and nociceptive effects, respectively [146–149]. Interestingly, dural nociceptors in female rodents show heightened sensitization in response to CGRP [150] and, prolactin has been reported to sensitize them for increased CGRP release [151, 152]. While this framework is bolstered by multiple lines of evidence, there remain outstanding questions that require clarification through future research. These include a deeper understanding of the involved molecular pathways and cell types, as well as the mechanisms that render them noxious, as inflammatory reactions in the brain are not always associated with headaches. Central pain-regulating mechanisms, as well as a migraine-specific genetic background, may modulate these mechanisms in inhibitory as well as facilitatory directions.

## Data Availability

No datasets were generated or analysed during the current study.

## Abbreviations

BBB: Blood-brain barrier
CAPS: Cryopyrin-associated periodic syndromes
CGRP: Calcitonin gene-related peptide
COX: Cyclooxygenase
CSD: Cortical spreading depolarization
CSF: Cerebrospinal fluid
HMGB1: High mobility group box protein 1
IĸB: Inhibitory kappa B
IL: Interleukin
iNOS: Inducible nitric oxide synthase
MS: Multiple sclerosis
NF-κB: Nuclear factor-kappa B
NLR: Nod-like receptor
NMDA: N-methyl-D-aspartate
NO: Nitric oxide
nNOS: Neuronal nitric oxide synthase
Panx: Pannexin
PET: Positron emission tomography
RAGE: Receptor for advanced glycation end products
TGF-β: Transforming growth factor beta
TLR: Toll-like receptor
TNF: Tumor necrosis factor
TSPO: 18 kDa translocator protein
